# Extraction of Silicon-Containing Nanoparticles from an Agricultural Soil for Analysis by Single Particle Sector Field and Time-of-Flight Inductively Coupled Plasma Mass Spectrometry

**DOI:** 10.3390/nano13142049

**Published:** 2023-07-11

**Authors:** Zhizhong Li, Madjid Hadioui, Kevin J. Wilkinson

**Affiliations:** Department of Chemistry, Université de Montréal, 1375 Ave. Thérèse-Lavoie-Roux, Montreal, QC H2V 0B3, Canada

**Keywords:** SP ICP-MS, SP ICP-TOF-MS, silica, Si-containing nanoparticles, pyrophosphate, soil

## Abstract

The increased use of silica and silicon-containing nanoparticles (Si-NP) in agricultural applications has stimulated interest in determining their potential migration in the environment and their uptake by living organisms. Understanding the fate and behavior of Si-NPs will require their accurate analysis and characterization in very complex environmental matrices. In this study, we investigated Si-NP analysis in soil using single-particle ICP-MS. A magnetic sector instrument was operated at medium resolution to overcome the impact of polyatomic interferences (e.g., ^14^N^14^N and ^12^C^16^O) on ^28^Si determinations. Consequently, a size detection limit of 29 ± 3 nm (diameter of spherical SiO_2_ NP) was achieved in Milli-Q water. Si-NP were extracted from agricultural soil using several extractants, including Ca(NO_3_)_2_, Mg(NO_3_)_2_, BaCl_2_, NaNO_3_, Na_4_P_2_O_7_, fulvic acid (FA) and Na_2_H_2_EDTA. The best extraction efficiency was found for Na_4_P_2_O_7,_ for which the size distribution of Si-NP in the leachates was well preserved for at least one month. On the other hand, Ca(NO_3_)_2_, Mg(NO_3_)_2_ and BaCl_2_ were relatively less effective and generally led to particle agglomeration. A time-of-flight ICP-MS was also used to examine the nature of the extracted Si-NP on a single-particle basis. Aluminosilicates accounted for the greatest number of extracted NP (~46%), followed by NP where Si was the only detected metal (presumably SiO_2_, ~30%).

## 1. Introduction

Si-containing nanoparticles (Si-NP), including silica (SiO_2_), are being used in a large number of modern applications, including paints [1,2], semiconductors [3,4], food additives [5,6], cosmetics [7,8] and medications [9,10]. With so many applications, there is expected to be a greater export of Si-containing NPs to the environment, potentially leading to their biouptake and harmful effects on organisms. To evaluate the potential risk of NPs, it is necessary to determine *biological effects*, which are related to NP composition, and *exposure*, which is determined by NP concentration and fate [11,12]. Since the properties of the NPs are largely linked to their size, particle size distributions also provide critical information on the potential bioavailability and the fate of the NP [13]. While there are several methods to characterize and quantify NPs, e.g., differential centrifugal sedimentation (DCS), nanoparticle tracking analysis (NTA), dynamic light scattering (DLS), static light scattering (SLS), scanning electron microscopy (SEM), transmission electron microscopy (TEM), small angle x-ray scattering (SAXS), field flow fractionation (FFF) [7,14,15], most of these techniques require high particle concentrations, long acquisition times or cannot distinguish among different chemical compositions.

SP ICP-MS is an extremely sensitive (low concentrations, small sizes) technique for analyzing inorganic nanoparticles [16,17,18,19,20]. Recent improvements to the technique, such as the use of desolvators [21], magnetic sector instruments [22], ion exchange resins [23,24], extremely short dwell times [22] or microdroplet injection [25,26] have further lowered the size detection limits of particles that can be accurately measured.

Nonetheless, the measurement of SiO_2_ NP by SP ICP-MS or indeed Si by ICP-MS is generally subject to spectral interferences resulting from various combinations of carbon, oxygen, and nitrogen (e.g., ^14^N^14^N^+^, ^12^C^16^O^+^, ^13^C^15^N^+^ for ^28^Si^+^; ^13^C^16^O^+^, ^14^N^15^N^+^, ^12^C^17^O^+^ for ^29^Si^+^; ^14^N^16^O^+^, ^18^O^12^C^+^, ^17^O^13^C^+^ for ^30^Si^+^). Several approaches have been used to eliminate or reduce polyatomic interferences. For example, for quadrupole-based instruments, CH_3_F and H_2_ can be added to a dynamic reaction cell [16,17,27,28,29,30]. The combination of Si and F leads to the formation of SiF^+^ that can be monitored at 44 amu, whereas H_2_ reacts with CO^+^ and N_2_^+^ (formation of ^12^C^16^O^1^H^+^ and ^14^N^14^N^1^H^+^, respectively) to allow the measurement of ^28^Si^+^, without spectral interferences. These approaches resulted in a size detection limit of about 80 nm for SiO_2_ NPs. Oxygen has also been used for Si analysis, leading to the formation of SiO_2_^+^ that can be monitored at 60, 61 or 62 amu [27].

The goal of this paper was to measure Si-based NP and colloids in soil. This is a difficult analytical problem for many reasons, including the high background of the soil, its heterogeneity, the inefficiency of the extraction procedures, and the difficulties mentioned above in measuring Si isotopes. A double-focusing magnetic sector field ICP-MS (ICP-SF-MS) was employed to avoid major interferences and detect the smallest Si-containing NPs possible. Furthermore, particles were isolated using a carefully optimized extraction procedure, which was designed to maximize the number of leached Si-NP, while reducing their aggregation and ensuring their stability. Si-containing NPs in soil leachates were analyzed under different conditions of ionic strength and natural organic matter content of the extraction solutions.

## 2. Materials and Methods

### 2.1. Chemicals

Ultrapure nitric acid (67–70%, Plasma Pure Plus) was purchased from SCP-Science (Montreal, QC, Canada). ACS reagent grade Ca(NO_3_)_2_, BaCl_2_, Na_2_H_2_EDTA and Na_4_P_2_O_7_ were purchased from Sigma-Aldrich (St. Louis, MO, USA). NaNO_3_ (>99%) was obtained from Fisher Scientific (Waltham, MA, USA), and Mg(NO_3_)_2_ (>99%) was provided by Fluka (Buchs, Switzerland). A standard fulvic acid (Suwannee River fulvic acid standard II, 2S101F) was obtained from the International Humic Substances Society (Denver, CO, USA).

Instrument sensitivities for ^197^Au and ^28^Si (used to convert signal intensity into analyte mass) were determined using a series of ionic standards (CGAu1, CGSi1) purchased from Inorganic Ventures (Christianburg, VA, USA) and prepared in 2% *v*/*v* HNO_3_ for Si (1, 2, 5, 10, 20 and 50 μg L^−1^) and 1% *v*/*v* HCl for Au (0.02, 0.05, 0.1, 0.2, 0.5 and 1 μg L^−1^). Suspensions of ultra-uniform gold nanoparticles (30 nm, 50 nm, 100 nm; NanoComposix, San Diego, CA, USA) were used to determine transport efficiency. For multi-element single particle analyses, sensitivity calibrations (0.2, 1, 5, 10, and 20 μg L^−1^ of each analyte in 1% *v*/*v* HNO_3_) were built with multi-element standards (a mixture of IV-ICPMS-71A, CGSi1 and CGTi1 from Inorganic ventures, Christianburg, VA, USA). Suspensions of SiO_2_ NP (nominally 80 nm and 200 nm) used for method validation were purchased from NanoComposix (San Diego, CA, USA). All extraction solutions and calibration standards were prepared using ultrapure water (Milli-Q; R > 18.2 MΩ cm; total organic carbon < 2 μg L^−1^).

### 2.2. Leaching of the Nanoparticles from the Soil

A soil sample was collected from an agricultural site of the Macdonald campus at McGill University [31]. Details on the principal physicochemical parameters are summarized in Table S1. Extractions of the nanoparticles from the soil were carried out by optimizing a standard leaching procedure developed previously for the total extraction of inorganic elements [32]. Different extraction media (Table 1) were examined for their capacity to separate nanoparticles while limiting particle dissolution and agglomeration. In each case, 10 mL of solution were added to 0.5 g of soil in a 15 mL polypropylene tube (SCP-Science DigiTUBEs). A continuous and gentle mixing was performed by rotating the tubes at 30 rpm for 18 h (Tube rotator 05-450-200, Fisher Scientific). The leachate was then separated from the solid phase by centrifugation (3000 rpm, 1882× *g*; 5 min; Heraeus multifuge 1 S-R, Kendro, Langenselbold, Germany). The collected supernatant was adequately diluted with ultrapure water before SP ICP-MS analysis. All leaching tests were conducted in triplicate.

### 2.3. Single Particle ICP-MS Analyses

SP ICP-MS measurements were performed on a double focusing inductively coupled plasma mass spectrometer (AttoM ES, Nu Instruments, Wrexham, UK) at medium resolution (2500), using the fast single ion acquisition mode and an optimized dwell time of 40 μs. SP ICP-MS data were processed offline using the Nu Quant software (version 2.1.2273.2, Nu Instruments, Wrexham, UK). Triplicate acquisitions, each of which lasted 40 s (i.e., 1 million data points), were recorded for each sample. For comparative purposes, size calculations were performed under the assumption that the Si-containing NPs were spherical SiO_2_ nanoparticles with a density of 2.65 g cm^−3^. Error bars represent standard deviations determined from triplicate measurements, rather than sample polydispersities.

A standard sample introduction system, consisting of a micro-flow concentric glass nebulizer (self-aspiration rate of 200 μL min^−1^ for 1 L min^−1^ Ar gas), a quartz cyclonic spray chamber (cooled to 4 °C), and a 1.5 mm quartz injector, was used to introduce the sample into the plasma. The mass transfer efficiency was determined from a suspension of ultra-uniform 30 nm gold nanoparticles (nanoComposix, San Diego, CA, USA) of known concentration (50 ng L^−1^) (Figure S1a). This allowed accurate determination of the actual sample flow rate reaching the plasma, which is used to calculate particle size and concentration. The mass transfer efficiency depends on the daily optimized instrument parameters and ranges from 0.20 to 0.26 μL s^−1^. In addition, a suspension of monodisperse spherical gold nanoparticles (50 nm, nanoComposix, San Diego, CA, USA) (Figure S1b) was analyzed daily as a quality control to validate instrument parameters for single particle mode. Measured sizes were 98–104% of the expected value, whereas recoveries for the particle number concentrations were 95–112%. Unfortunately, no certified SiO_2_ NPs were available for additional quality control; however, two SiO_2_ NP suspensions of known mean sizes (nominally 80 nm and 200 nm) were systematically analyzed to validate the analytical parameters further.

Although low resolutions are generally used to achieve the best sensitivities and thus the lowest size detection limits, the use of a medium resolution (2500) was used for ^28^Si to eliminate interferences resulting from combinations of the most abundant isotopes of carbon (^12^C), oxygen (^16^O) and nitrogen (^14^N) (Figure S2). Consequently, at 28 amu, lower background noise was obtained, leading to well-resolved peaks of small SiO_2_ nanoparticles (Figure S3). Peak masses were determined from their intensities following calibration from 0–50 μg L^−1^ (Figure S4). In addition, several additional strategies were evaluated to improve the sensitivity (Table S2). For example, while using a desolvator did improve sensitivity [21,28], it came with a substantial increase in the background noise. Indeed, the signal to noise (noise determined from 2% *v*/*v* HNO_3_) evaluated for 10 μg L^−1^ Si was higher for the wet aerosol (6.7 ± 2.6) than for the dry aerosol (1.1 ± 0.1). The use of an alumina torch showed no significant improvement as compared to the quartz torch, suggesting that the torch material did not contribute substantially to the background noise. Several different polypropylene tubes [43] were also tested for contamination in order to use those with the lowest Si impurities (DigiTUBEs, SCP Science, Baie-d’Urfé, QC, Canada).

SP ICP-TOF-MS measurements were performed on a high-speed time-of-flight ICP-MS (Vitesse, Nu Instruments, UK) using a standard sample introduction similar to the one used for SP ICP-MS measurements, except for the glass concentric nebulizer which had a self-aspiration rate of 400 μL min^−1^ for 1 L min^−1^ Ar gas. SP ICP-TOF-MS data were acquired in triplicate for 92 s with a dwell time of 76.8 µs. Data were processed by Nu Quant Vitesse (version 1.2.7893.1, Nu Instruments, UK).

## 3. Results and Discussion

### 3.1. Choice of the Best Extractant

**Figure 1:**
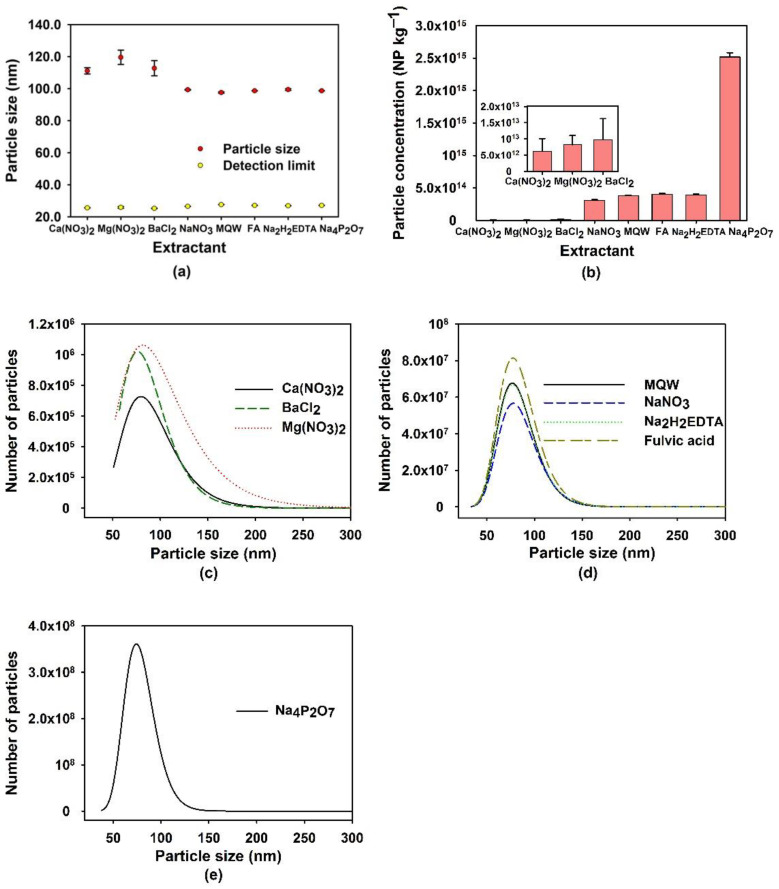
(**a**) Average sizes, (**b**) number concentrations and (**c**–**e**) size distributions of Si-containing NPs in the extraction solutions after 18 h of contact with different extractants (Milli-Q water (MQW) or 5 mmol L^−1^ of Ca(NO_3_)_2_, Mg(NO_3_)_2_, BaCl_2_, NaNO_3_ or Na_4_P_2_O_7_; 0.1 mmol L^−1^ for Na_2_H_2_EDTA; 40 mg L^−1^ of fulvic acid). For the size distributions (**c**–**e**), the leachates were diluted 40,000 times before SP ICP-MS analysis, except for Na_2_P_2_O_7,_ where a dilution factor of 250,000 times was required. Particle sizes correspond to equivalent diameters calculated, assuming that the NP are spherical SiO_2_.

The choice of the extraction media for the soil was guided by the number of particles brought into the solution and by the resulting particle size distribution (Figure S5) by considering extractant purity (i.e., blank levels, Figure S6). Sizes and concentrations of the extracted Si-containing NPs are shown in Figure 1. The use of the divalent salts: Ca(NO_3_)_2_, Mg(NO_3_)_2_ and BaCl_2_ [44] led to the largest average diameters (115 ± 5 nm, Figure 1a) and the lowest number concentrations ((8 ± 4) × 10^12^ kg^−1^, Figure 1b). All other extractants gave similar average particle sizes of 98.7 ± 0.7 nm. With respect to the concentration of Si-containing NPs, the most efficient leaching reagent was Na_4_P_2_O_7_, which extracted five times more NPs than pure water, NaNO_3_, fulvic acid or EDTA and 200× more than the Ba^2+^, Ca^2+^ or Mg^2+^ salts.

The extraction agents and the NPs are subject to various physicochemical interactions. Indeed, soil grains (composed largely of silicates) contain numerous pores of different sizes and shapes, through which particles and dissolved species may be exchanged with the surrounding media [45,46,47]. Si tetrahedrons (SiO_4_^4−^) can be present in the soil as isolated units (dissolved), or they may form single or double polymer chains or bands, layers or three-dimensional networks [45]. Some of these polymers could be sufficiently large to be detected as NPs. Furthermore, aluminosilicates are ubiquitous in the soil. Indeed, due to a structural charge deficit, the aluminosilicates are generally negatively charged, which increases their colloidal stability. The addition of the divalent cations (e.g., Ca^2+^, Mg^2+^, Ba^2+^) could thus destabilize the colloidal aluminosilicates, either by charge screening or by a cation bridging of the particles, resulting in agglomeration and leading to larger overall particle sizes [47,48]. Indeed, Loosli et al. have demonstrated that Ca^2+^ can adsorb to the surface of particles, resulting in a reduction in negative surface potential and the formation of large agglomerates [49]. The low extraction efficiency observed when using the divalent cations (Figure 1) is likely related to the formation of these agglomerates, leading to lower particle numbers and larger overall sizes (Table S3).

No noticeable differences in NP numbers or sizes were observed following extraction by NaNO_3_ or Milli-Q water. The lack of induced agglomeration is consistent with the absence of multivalent cations in the extraction solutions. Similarly, the EDTA and the fulvic acid extracted similar numbers of similarly sized Si-containing NPs. Since both are strong ligands for divalent (and multivalent) cations, the observations may be due to the complexation of potentially destabilizing cations in the solution. Fulvic acids are also known for their stabilizing effect on colloidal systems [48] via increased electrostatic repulsion of the colloidal surfaces, which may also have contributed in that case. For example, Yan et al. demonstrated that humic acids present in groundwater could not enhance metal complexation if the soil was not saturated with humic acid [50]. In other words, the humic acids had a greater propensity to bind the particle surface than the metals in the solution. Pyrophosphate (P_2_O_7_^4−^) can also chelate cations in solution and be adsorbed to the particle surfaces, both actions which could increase NP stabilization in solution [49] through an increase in the negative zeta potential (Table S4) and a decrease in particle screening. Indeed, the highest number of small particles in the leachate was obtained when Si-containing NPs were extracted by the Na_2_P_2_O_7_ (Figure 1e).

**Figure 2:**
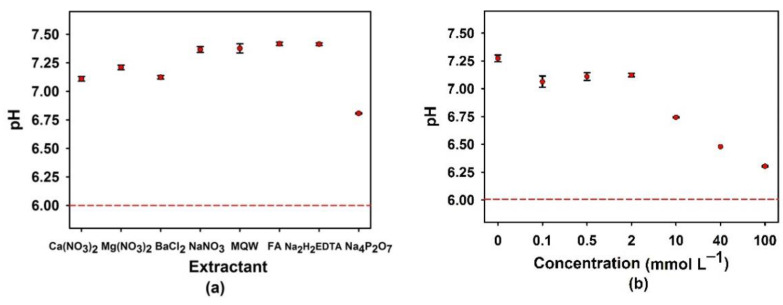
pH of the extraction solutions after an 18-h contact of the soil with (**a**) different extractants and (**b**) different concentrations of Na_4_P_2_O_7_. pH was initially adjusted to 6.0 (--- in the figure) before the start of the extraction. The concentrations of the extractants in (**a**) were: 5 mmol L^−1^ for Ca(NO_3_)_2_, Mg(NO_3_)_2_, BaCl_2_, NaNO_3_ and Na_4_P_2_O_7_, 0.1 mmol L^−1^ for Na_2_H_2_EDTA, and 40 mg L^−1^ for FA.

In all cases, the extraction solutions were adjusted to pH 6.0, immediately before adding the soil and the pH was measured again after the 18 h extraction. The observed deviation from pH 6.0 depended on the nature of the extractant (Figure 2a) and was related to its buffer capacity and that of the soil. In all cases, the pH increased, probably due to the dissolution of soil carbonates. Extractants with the weakest buffer capacity (pure water, fulvic acid and EDTA) had the largest pH shifts (from 6.0 to 7.4), whereas the smallest pH change (6.0 to 6.8) was observed for Na_4_P_2_O_7_ (Figure 2a). Furthermore, higher Na_4_P_2_O_7_ concentrations had smaller pH variations (Figure 2b), consistent with the work of Zhao et al. [51], who found different pH after leaching for different extractants to soil ratios, during their extractions with ethylenediamine disuccinic acid (EDDS) and FeCl_3_.

### 3.2. Optimisation of the Na_4_P_2_O_7_ Extraction Conditions

**Figure 3:**
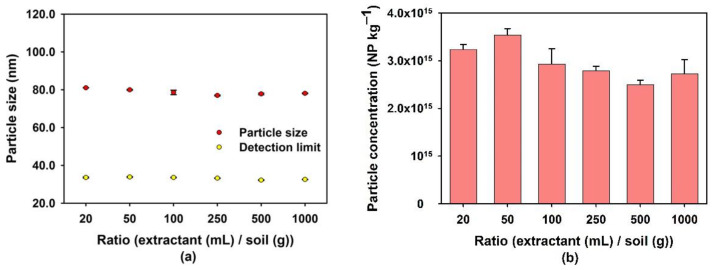
(**a**) Average size and (**b**) concentration of Si-containing NPs being leached from the soil as a function of the ratio of the volume of the extractant (mL) to the mass of soil (g). The concentration of the extractant (Na_4_P_2_O_7_) was 40 mmol L^−1^ and its pH was 6.0. Experiments were performed on soil that was sampled from the McGill MacDonald Campus. Particle sizes correspond to equivalent diameters calculated under the assumption that the NP are spherical SiO_2_.

The impact of the liquid/solid ratio (expressed as the volume of the extraction solution with respect to the mass of soil) was studied using 40 mM Na_4_P_2_O_7_ as the extractant (Figure 3). This ratio had very little impact on the extraction of Si-containing NPs, likely because the concentration of Na_4_P_2_O_7_ was already optimal, even for the lowest ratio considered here. The impact of this ratio on the extraction process is influenced by several parameters, including the nature of the soil, the type of extractant and the target species. For example, Navarro-González et al. [52] found that an increase in the ratio of extractant (mL) to sludge (g) from 3 to 50 significantly decreased the extraction of heavy metals. On the other hand, Hall and Pelchat [53] reported minimal variation in the amount of C, Hg or Zn leached from soils when the ratio of extractant volume (mL) to soil mass (g) ranged from 10 to 200. Li et al. [54] found that the number of particles extracted by pyrophosphate increased slightly as the liquid/solid ratio varied from 5 to 40 and then decreased above 80; a tendency similar to the results shown in Figure 3b. Although it appears (Figure 3b) that a ratio of 50 led to the highest extraction of Si-containing NPs, there was, in fact, no significant difference (*p* = 0.183) for the number of extracted Si-containing NPs, when comparing the ratios of 20 and 50. Therefore, a ratio of 20 was used for subsequent experiments, corresponding to the largest soil mass (0.5 g), which helped reduce errors related to soil inhomogeneity.

**Figure 4:**
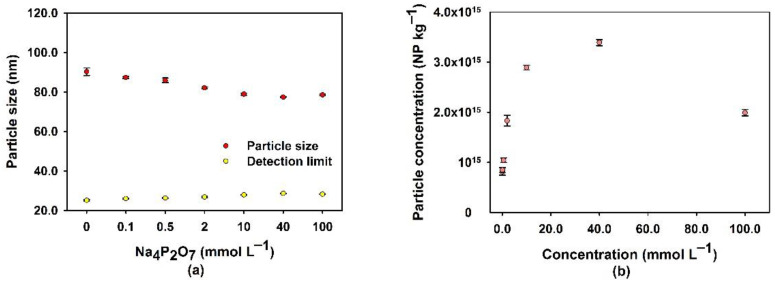
(**a**) Average size and (**b**) concentration of Si-containing NPs in the Na_4_P_2_O_7_ extraction solution as a function of its concentration after an 18-h contact time. Experiments were performed on soil that was sampled from the McGill MacDonald Campus. Particle sizes correspond to equivalent diameters calculated under the assumption that the NP are spherical SiO_2_.

The role of Na_4_P_2_O_7_ concentration was also studied by varying its concentration from 0.1 to 100 mmol L^−1^ (Figure 4). As Na_4_P_2_O_7_ was increased from 0.1 to 40 mM, the average size of the extracted Si NPs decreased from 90 nm to 80 nm (Figure 4a), and their number concentration increased from 0.8 × 10^15^ kg^−1^ to 3.4 × 10^15^ kg^−1^ (Figure 4b). This observation is consistent with the plausible role of P_2_O_7_^4−^ in decreasing agglomeration, as discussed above.

Finally, for some of the other extractants, the role of concentration was also examined. For example, the Ca(NO_3_)_2_ and NaNO_3_ concentrations varied from 0.1 to 10 mmol L^−1^, and the concentration of FA varied between 0.2 and 50 mg L^−1^ (Figure S7). While few differences were observed in either NP numbers or sizes for NaNO_3_ and FA over these concentration ranges, increased Ca^2+^ clearly impacted the extraction efficiency. Indeed, as the concentration of Ca(NO_3_)_2_ was increased from 0.1 to 5 mmol L^−1^, a slight increase in average particle size (Figure S7a) and an abrupt drop in the number of extracted Si NPs (Figure S7d) were observed. These observations were attributed to increased agglomeration leading to sedimentation of the larger structures.

### 3.3. Stability of the Extracted NP with Time

**Figure 5:**
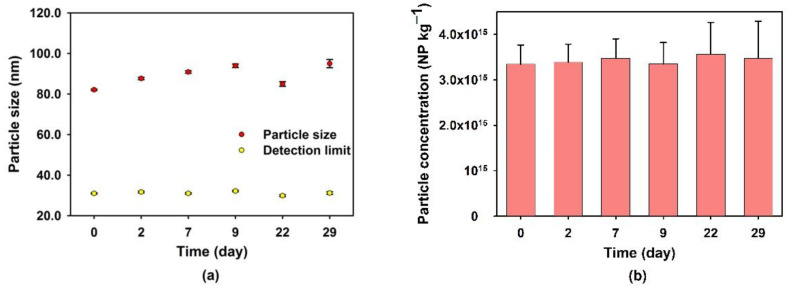
(**a**) Average size and (**b**) concentration of the Si-containing NPs in the soil extraction solution as a function of time. The extraction of nanoparticles was carried out using a solution of 40 mmol L^−1^ Na_2_P_2_O_7_. Particle sizes correspond to equivalent diameters calculated under the assumption that the NP are spherical SiO_2_.

The stability of the extracted NPs was examined by first extracting the particles from the soil using Milli-Q water and then suspending them in the different media (Ca(NO_3_)_2_, BaCl_2_, Na_4_P_2_O_7_, or EDTA). The suspensions were gently mixed (rotation) for 24 h at room temperature before SP ICP-MS analysis. Based upon an increase in the average particle sizes and a decrease in the particle number concentrations (when compared to Milli-Q water), Ba^2+^ and Ca^2+^ appeared to induce agglomeration (Figures S8 and S9). For its part, EDTA did not result in a change of either particle size or number concentration. For Na_4_P_2_O_7_, a slight decrease in particle size and an increase in particle number concentration indicates, as previously stated, that the P_2_O_7_^4−^ plays a role in breaking down the particle agglomerates. Pyrophosphate also seemed to stabilize the nanoparticles over time, such that only small size and number concentration variations were discerned following one month of storage at 4 °C in the dark (Figure 5).

### 3.4. Nature of the Si-Containing NP

**Figure 6:**
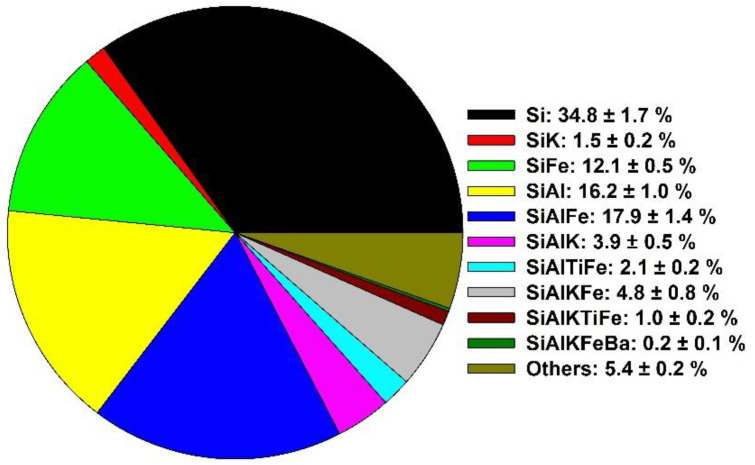
The percentage of each type of NP (based on the number of nanoparticles) in a leachate solution from the McGill McDonald Campus soil using 40 mM pyrophosphate as the extractant. Results were obtained using SP ICP-TOF-MS. The leachates were diluted 100,000 times before analysis. Results are determined from the analysis of the 1967 Si-containing NP.

Finally, the nature of the extracted NP was examined, on a single particle basis, using SP ICP-TOF-MS on the pyrophosphate extracted NP (Figure 6). SP ICP-TOF-MS simultaneously measures multiple elements for a given NP. A significant proportion of the NP could be classified as aluminosilicates as at least 46% of the detected NP contained Si and Al, in addition to other elements such as K, Fe or Ti, in varying proportions. Si NP containing no other detected elements were found in 34.8% of the NP. However, these NP were generally smaller (average Si mass of 5.4 ± 0.5 fg compared to 7.0 ± 0.1 fg for the SiAlFe NP) and nearer the size detection limits of the SP ICP-TOF-MS. This implies that 34.8% is an upper limit of the SiO_2_ NP, as secondary elements may simply have not been detected. Indeed, when Milli-Q water was used to extract the NP (Figure S10), Si was the only element detected in only 18.8% of the particles. The relatively larger proportion of multielement Si-containing NP that were extracted by water as compared to Na_4_P_2_O_7_ could not be attributed to better mass detection limits of K, Fe, Al, Ti, and Ba, as detection limits were actually similar in the two media. Indeed, as the extracted samples were diluted 100,000× before their measurements by SP ICP-TOF-MS, very low background noise was obtained. This near absence of differences among the detection limits suggests that in addition to a greater number of particles being extracted by the Na_4_P_2_O_7_, on average, the particles were smaller (Figure 4a, Table S4), with relatively more SiO_2_ and relatively fewer aluminosilicates (though not fewer aluminosilicates in absolute terms). These data support our contention that pyrophosphate was the most appropriate extractant of Si NP (both anthropogenic and natural) from the soil. Nonetheless, the use of Milli-Q water could be justified when studying natural processes such as the (more gentle) mobilization of soil colloids due to their interaction with rain or subsurface waters [55].

Figure 6 shows the analysis of the Si-containing NP. When all particle types are considered, Si-containing NP accounted for about half of the particles detected in this soil, while Fe-containing NP comprised another significant fraction (30% of the NP were Fe NP and this value increased to 57.8% when other elements, including Si, were included in the calculations). Different NP types were also detected but in smaller numbers.

## 4. Conclusions

Silicon is everywhere in the environment (particularly in very large proportions in soil) as aluminosilicates or SiO_2_, among other forms. Its analysis by ICP-MS is difficult, especially due to polyatomic interferences (e.g., ^14^N^14^N and ^12^C^16^O for ^28^Si). The use of an ICP-SF-MS helps to reduce these interferences and, hence, lower size detection limits for single particle analysis. Indeed, using a sector field instrument at medium resolution, a size detection limit of 29 ± 3 nm was obtained for SiO_2_, which contrasts well with detection limits of ~80 nm currently reported in the literature using quadrupole-based instruments. Future work on lowering size detection limits for the multi-element ICP-TOF-MS is necessary to ensure that the compositions of the smallest NP are determined accurately. Among the different extractants investigated here, Na_4_P_2_O_7_ was the most efficient for leaching the largest numbers of Si-NP from soil and giving the most stable suspensions for SP analysis. Concentration played an important role in the extraction process for which the best results were obtained for 40 mM Na_4_P_2_O_7_. Extraction solutions of Ca(NO_3_)_2_, Mg(NO_3_)_2_ or BaCl_2_ were less effective and led to agglomeration of the Si-NP in the leachates. Water was shown to be a useful extractant for colloidal particles easily mobilized in the soils. Finally, mainly silicates and aluminosilicates were extracted from the agricultural soil, though the chemical heterogeneity of the particles suggests that other NP were equally extracted.

## Figures and Tables

**Table 1:** Extraction media tested for the leaching of the Si-containing NPs. The pH was adjusted to 6.0 for all extraction solutions.

Composition	Concentration	References
IHSS standard fulvic acid (FA)	40 mg L^−1^	[33,34]
Na_2_H_2_EDTA	0.1 mM (40 mg L^−1^)	[35,36,37]
Ca(NO_3_)_2_	5 mM	[35,38,39,40]
Mg(NO_3_)_2_	5 mM	[36,40]
NaNO_3_	5 mM	[39,40,41]
BaCl_2_	5 mM	[37,42]
Na_4_P_2_O_7_	5 mM	[37,41,42]

## Data Availability

All raw data are available from the authors upon reasonable request.

## Supplementary Materials

The following supporting information can be downloaded at: https://www.mdpi.com/article/10.3390/nano13142049/s1, Figure S1: (a) Signal intensity distribution of the suspension of 30 nm ultra-uniform gold nanoparticles used for transport efficiency determination and (b) particle size distribution of the 50 nm Au NP standard that was analyzed for quality control; Figure S2: Magnet scan of ^28^Si at medium resolution (2500). The concentration of Si was 20 μg L^−1^; Figure S3: Time-resolved signal for (a) a suspension of engineered SiO_2_ nanoparticles (80 nm nominal diameter) and (c) a soil leachate. Time-resolved signal of a small peak of SiO_2_ nanoparticle for (b) the suspension of engineered SiO_2_ nanoparticles (44.6 nm) and (d) the soil leachate (39.7 nm); Figure S4: Example of external 28Si calibration curve (ionic standard) used to determine Si masse (particulate and dissolved); Figure S5: Particle size distribution of Si-containing NPs in the soil leachates obtained using different extractants: (a) Ca(NO_3_)_2_ (5 mmol L^−1^), (b) BaCl_2_ (5 mmol L^−1^), (c) Mg(NO_3_)_2_ (5 mmol L^−1^), (d) Milli-Q water, (e) NaNO_3_ (5 mmol L^−1^), (f) Na_2_H_2_EDTA (0.1 mmol L^−1^), (g) FA (40 mg L^−1^) and (h) Na_4_P_2_O_7_ (5 mmol L^−1^); Figure S6: Raw data for SP ICP-MS analysis of ^28^Si in (a) Milli-Q water, (b) solution of 40 mM Na_4_P_2_O_7_ that was diluted 250,000 times before analysis, (c) diluted (250,000×) soil leachate obtained using Milli-Q water, and (d) diluted (250,000×) soil leachate obtained with 40 mM Na_4_P_2_O_7_. All dilutions were done with Milli-Q water; Figure S7: (a–c) size and (d–f) concentration of Si-containing NPs in the extraction solutions of soil in contact (18 h) with different extractants as a function of the concentration of (a,d) Ca(NO_3_)_2_, (b,e) NaNO_3_, and (c,f) FA; Figure S8: Particle size distribution of Si-containing NPs extracted from the soil using Milli-Q water, which was then suspended in different media: (a) Ca(NO_3_)_2_ (5 mmol L^−1^), (b) BaCl_2_ (5 mmol L^−1^), (c) Milli-Q water, (d) Na_2_H_2_EDTA (0.1 mmol L^−1^) or (e) Na_4_P_2_O_7_ (5 mmol L^−1^). (f) Log Normal fitting the particle size histograms; Figure S9: (a) Average size and (b) concentration of Si-containing NPs extracted from the soil using Milli-Q water, which was then suspended in different media: Ca(NO_3_)_2_ (5 mmol L^−1^), BaCl_2_ (5 mmol L^−1^), Na_4_P_2_O_7_ (5 mmol L^−1^) or Na_2_H_2_EDTA (0.1 mmol L^−1^); Figure S10: Proportion of each Si particle type detected in the soil leachate using pure water as the extractant and SP ICP-TOF-MS for detection. Leachates were diluted 100,000 times before analysis. Eight hundred fifty-four particles were detected during an acquisition time of 92 s. A dwell time of 76.8 µs was employed: Table S1: Physicochemical parameters of the McGill MacDonald College soil sample; Table S2: Results comparing the signal: noise (S/N) of the quartz and alumina torches in wet and dry mode of the instrument; Table S3: Percentage of Si-containing NPs below of different size for the different extractants; Table S4: Results for DLS (dynamic light scattering) analysis of the extracted NP.
