# Supplementary material for: Extraction of Silicon-Containing Nanoparticles from an Agricultural Soil for Analysis by Single Particle Sector Field and Time-of-Flight Inductively Coupled Plasma Mass Spectrometry
Source: Nanomaterials (Basel). 2023 Jul 11;13(14):2049. doi: 10.3390/nano13142049 (PMC10383646; doi:10.3390/nano13142049)
Supplement: Supplementary file 1 [file nanomaterials-13-02049-s001.zip › nanomaterials-2452181-supplementary.pdf]

nanomaterials

Supporting material for

Extraction of silicon-containing nanoparticles from an agricultural soil for analysis by single particle sector field and time-of-flight inductively coupled plasma mass spectrometry

Zhizhong Li [1], Madjid Hadioui [1], Kevin J. Wilkinson [1,*]

[1] Department of Chemistry, Université de Montréal, 1375 Ave. Thérèse-Lavoie-Roux
Montreal, QC, H2V 0B3;
* Correspondence: kj.wilkinson@umontreal.ca

List of tables:

- **Table S1.** Physicochemical parameters of the McGill MacDonald College soil sample.
- **Table S2.** Results comparing the signal: noise (S/N) of the quartz and alumina torches in wet and dry mode of the instrument. Also, two types of polypropylene tubes (PP and DigiTube) were tested for Si contamination. S/N were determined for 10 ug L^{-1} of a Si solution in 2% HNO_3. Signal intensities were determined for ^{28}Si using the medium resolution (2500) of instrument.
- **Table S3.** Percentage of Si-containing NPs below different size cut-offs for the different extractants.
- **Table S4.** Results for DLS (dynamic light scattering) of the extracted NP. All of the extractants were adjusted to pH 6.0 before mixing them with a suspension of SiO_2 NP or the soil. SiO_2 NP suspensions were diluted to 100 ppm using the extractant, while the soil was extracted for 18 h at 30 rpm and then diluted 10 times in the extractant before analysis, except for the $Ca(NO_3)_2$ extractant, which wasn't diluted. MQW= Milli-Q water.

List of figures:

- **Figure S1.** (a) Signal intensity distribution of the suspension of 30 nm ultra-uniform gold nanoparticles used for transport efficiency determination and (b) particle size distribution of the 50 nm Au NP standard that was analyzed for quality control.
- **Figure S2.** Magnet scan of ^{28}Si at medium resolution (2500). Concentration of Si was 20 μg L^{-1}.
- **Figure S3.** Time-resolved signal for (a) a suspension of engineered SiO_2 nanoparticles (80 nm nominal diameter) and (c) a soil leachate. Time-resolved signal of a small peak of SiO_2 nanoparticle for (b) the suspension of engineered SiO_2 nanoparticles (44.6 nm) and (d) the soil leachate (39.7 nm). The soil leachate was obtained using Milli-Q water as the extraction solution. The dwell time used was 40 μs.
- **Figure S4**. Example of external ^{28}Si calibration curve (ionic standard) used to determine Si mass (particulate and dissolved).
- **Figure S5.** Particle size distribution of Si-containing NPs in the soil leachates obtained using different extractants: (a) $Ca(NO_3)_2$ (5 mmol L^{-1}), (b) $BaCl_2$ (5 mmol L^{-1}), (c) $Mg(NO_3)_2$ (5 mmol L^{-1}), (d) Milli-Q water, (e) $NaNO_3$ (5 mmol L^{-1}), (f) Na_2H_2EDTA (0.1 mmol L^{-1}), (g) FA (40 mg L^{-1}) and (h) $Na_4P_2O_7$ (5 mmol L^{-1}). The pH was adjusted to 6.0 for all extraction solutions prior to contact with the soil. The leachates were diluted 40000 times prior to SP-ICP-MS analysis, except in the case of the $Na_4P_2O_7$ for which a dilution factor of 250000 times was necessary. Particle sizes correspond to equivalent diameters calculated under the assumption that the NP are spherical SiO_2.
- **Figure S6**. Raw data for SP ICP-MS analysis of ^{28}Si in a) Milli-Q water, b) solution of 40 mM $Na_4P_2O_7$ that was diluted 250000 times prior to analysis, c) soil leachate obtained using Milli-Q water and diluted (250000x), and d) soil leachate obtained with 40 mM $Na_4P_2O_7$ and diluted (250000x). All dilutions were performed using Milli-Q water.
- **Figure S7.** (a, b, c) size and (d, e, f) concentration of Si-containing NPs in the extraction solutions of a soil that was in contact (18 hours) with different concentrations of different extractants: (a, d) $Ca(NO_3)_2$, (b, e) $NaNO_3$, and (c, f) FA. The concentration of 0 in the extraction solutions represents an extraction using Milli-Q water. Particle sizes correspond to equivalent diameters calculated under the assumption that the NP are spherical SiO_2.

- **Figure S8.** Particle size distribution of Si-containing NPs extracted from the soil using Milli-Q water, which were then suspended in different media: (a) $Ca(NO_3)_2$ (5 mmol L^{-1}), (b) $BaCl_2$ (5 mmol L^{-1}), (c) Milli-Q water, (d) Na_2H_2EDTA (0.1mmol L^{-1}) or (e) $Na_4P_2O_7$ (5 mmol L^{-1}). (f) Polynomial fitting the particle size histograms. The leachates were diluted 100000 times prior to SP-ICP-MS analysis. Particle sizes correspond to equivalent diameters calculated under the assumption that the NP are spherical SiO_2.
- **Figure S9.** (a) Average diameters and (b) concentration of Si-containing NPs extracted from the soil using Milli-Q water, which were then suspended in different media: $Ca(NO_3)_2$ (5 mmol L^{-1}), $BaCl_2$ (5 mmol L^{-1}), $Na_4P_2O_7$ (5 mmol L^{-1}) or Na_2H_2EDTA (0.1 mmol L^{-1}). Particle sizes correspond to equivalent diameters calculated under the assumption that the NP are spherical SiO_2.
- **Figure S10**. Proportion of each Si particle type detected in the soil leachate using pure water as the extractant and SP TOF-ICP-MS for detection. Leachates were diluted 100000 times prior to analysis. 854 particles were detected during an acquisition time of 92 s. A dwell time of 76.8 µs was employed.

Table S2. Physicochemical parameters of the McGill MacDonald College soil sample [1]

**Physicochemical parameter**	
pH[a]	7.2
Organic matter (%)	6.1
P (mg kg^{-1})	183
Ca (mg kg^{-1})	3999
Mg (mg kg^{-1})	325
K (mg kg^{-1})	349
Al (mg kg^{-1})	717
N (NO^{3-}) (mg kg^{-1})	6.4
N (NH_4^+) (mg kg^{-1})	2.5

[a] A 1:1 (w/v) solution of soil to water was shaken for 30 min, then left to rest for 1 hour.

Table S2. Results comparing the signal: noise (S/N) of the quartz and alumina torches in wet and dry mode of the instrument. Also, two types of polypropylene tubes (PP and DigiTube) were tested for contamination for Si. S/N were given for 10 ug L^{-1} of a Si solution in 2% HNO_3. Signal intensities were determined for ^{28}Si using the medium resolution of instrument.

	**S/N**		**Signal (x 10^4cps)**	
	Quartz torch	Alumina torch	PP tube	Digi tube
Wet mode	6.7 ± 2.6	4.0 ± 1.5	20.8 ± 2.2	6.7 ± 0.1
Dry mode	1.1 ± 0.1	1.09 ± 0.02		

Table S3. Percentage of Si-containing NPs below different size cut-offs for the different extractants.

Extractant	NPs<70nm (%)	NPs<80nm (%)	NPs<90nm (%)	NPs<100nm (%)
$Ca(NO_3)_2$	17.5 ± 0.9	32.1 ± 2.6	45.0 ± 4.7	54.7 ± 4.4
$Mg(NO_3)_2$	13.2 ± 1.9	28.8 ± 1.4	39.6 ± 1.6	48.2 ± 0.6
$BaCl_2$	17.1 ± 2.2	36.4 ± 4.8	49.0 ± 4.1	59.2 ± 2.9
$NaNO_3$	19.8 ± 0.6	38.5 ± 0.8	53.2 ± 1.1	64.0 ± 0.9
MQW	21.5 ± 0.9	40.9 ± 1.0	55.4 ± 0.9	66.6 ± 0.6
FA	20.2 ± 0.9	39.5 ± 0.9	54.0 ± 0.9	64.8 ± 0.7
EDTA	20.8 ± 1.0	40.0 ± 1.3	54.5 ± 1.3	65.1 ± 1.4
$Na_4P_2O_7$	26.2 ± 0.7	48.1 ± 0.7	63.0 ± 0.9	73.7 ± 0.7

Table S4. Results for DLS (dynamic light scattering) of the extracted NP. All of the extractants were adjusted to pH 6.0 before mixing with the suspension of SiO_2 nanoparticles or the soil. The SiO_2 NP suspension was diluted to 100 ppm using the extractant, while the soil was extracted for 18 h at 30 rpm and then diluted 10 times using the extractant before analysis, except for the $Ca(NO_3)_2$, which wasn't diluted. MQW= Milli-Q water.

	**Extractant**	**Zeta Potential (mV)**			**Diameter (nm)**		
		Mean		SD	Mean		SD
	MQW	-32.6	±	2.0	87.0	±	0.7
SiO_2 nanoparticles	$Ca(NO_3)_2$	-18.0	±	0.4	86.9	±	8.9
(80 nm)	$Na_4P_2O_7$	-27.4	±	2.8	88.7	±	7.6
	Fulvic acid	-44.2	±	4.2	84.9	±	0.6
	MQW	-43.4	±	3.0	203.2	±	1.6
SiO_2 nanoparticles	$Ca(NO_3)_2$	-23.1	±	2.0	199.1	±	2.2
(200 nm)	$Na_4P_2O_7$	-33.5	±	1.3	199.7	±	0.9
	Fulvic acid	-49.1	±	2.7	201.5	±	2.4
	MQW	-17.1	±	1.0	243.4	±	2.5
Extracted soil nanoparticles	$Ca(NO_3)_2$	-12.6	±	0.2	792.4	±	46.4
	$Na_4P_2O_7$	-35.0	±	0.7	213.3	±	2.0
	Fulvic acid	-23.9	±	0.5	254.8	±	5.0

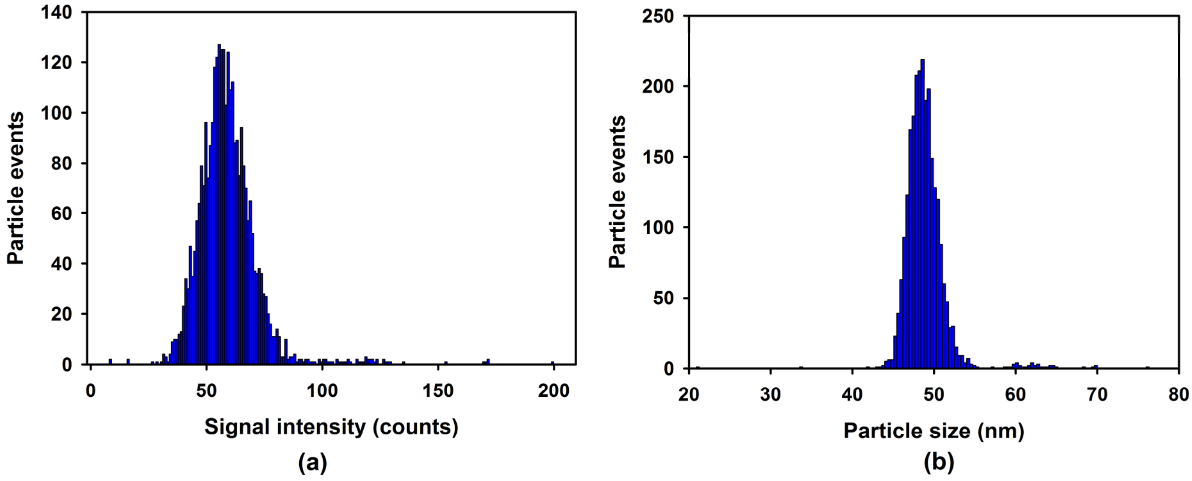

Figure S1. Measured particle distributions of the ultra-uniform gold nanoparticles for the (a) 30 nm and (b) 50 nm standard NP.

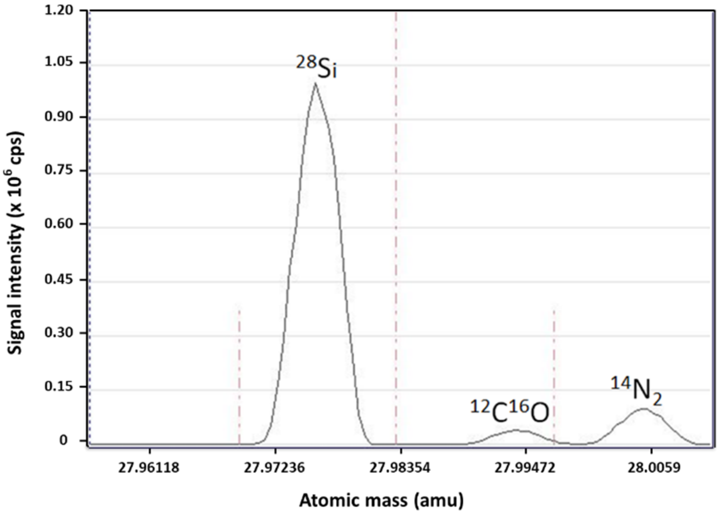

Figure S2. Magnet scan of ^{28}Si at medium resolution (2500). Si concentration was 20 μg L^{-1}.

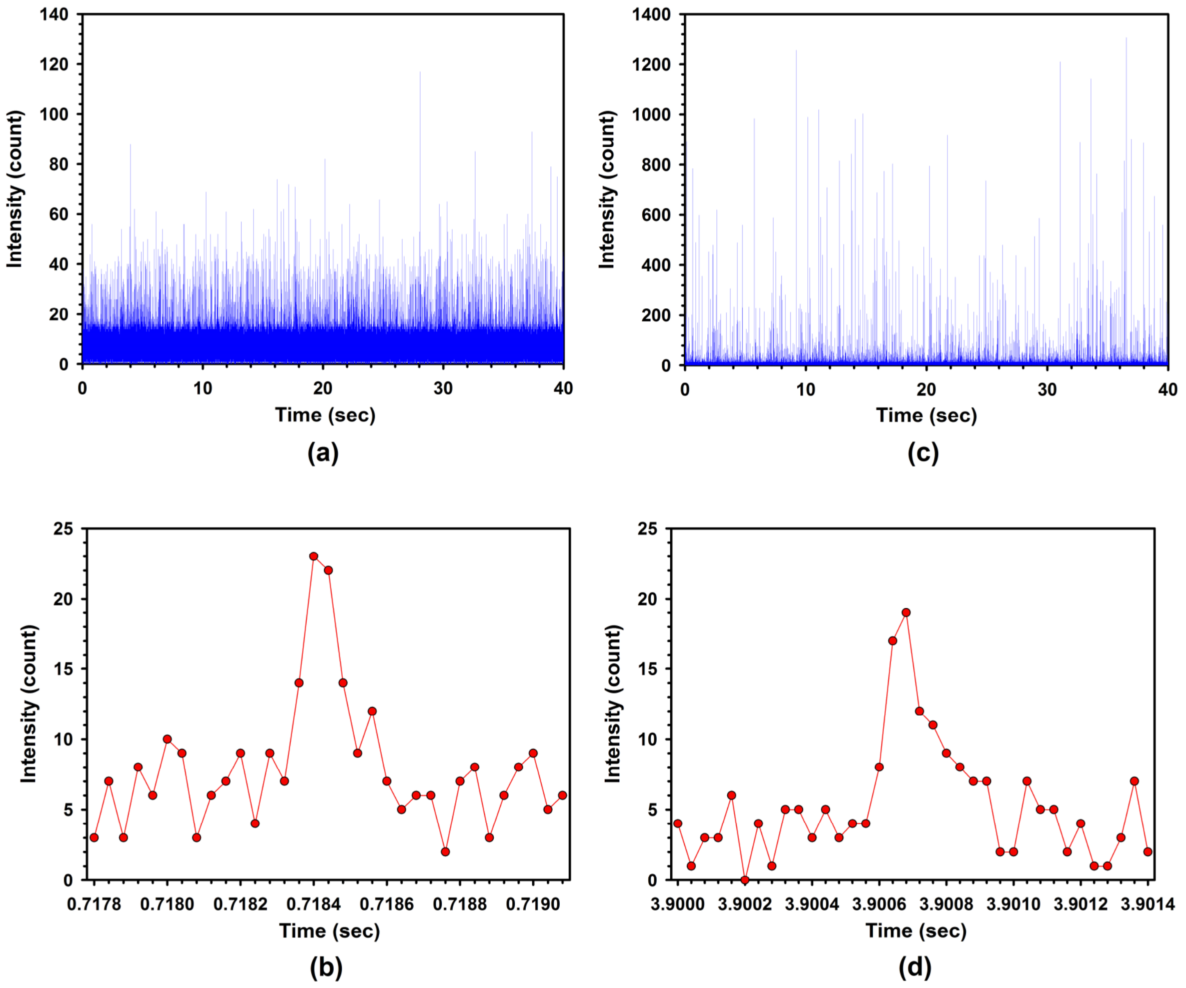

Figure S3. Time-resolved signal for (a) a suspension of engineered SiO_2 nanoparticles (80 nm nominal diameter) and (c) a soil leachate. Time-resolved signal of a small peak of SiO_2 nanoparticle for (b) the suspension of engineered SiO_2 nanoparticles (44.6 nm) and (d) the soil leachate (39.7 nm). The soil leachate was obtained using Milli-Q water as the extraction solution. The dwell time was 40 μs.

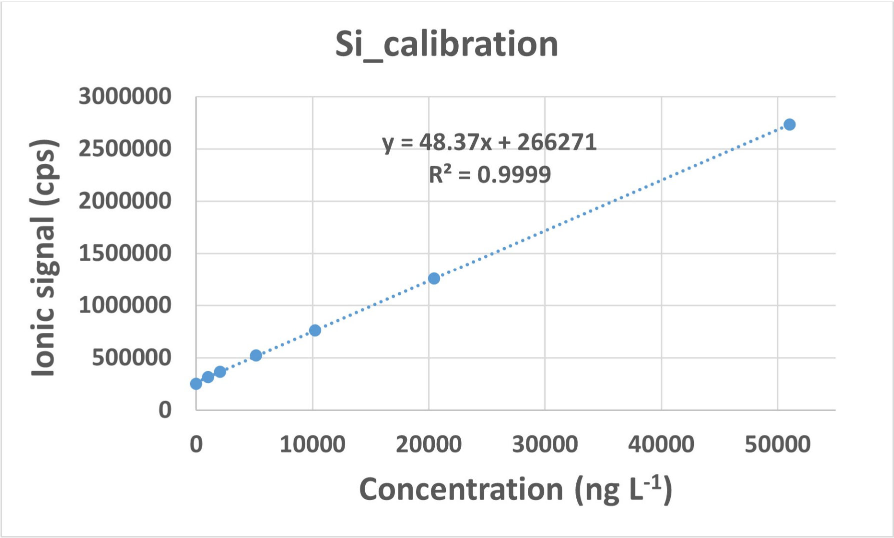

Figure S4. Example of external [28]Si calibration curve (ionic standard) used to determine Si mass (particulate and dissolved).

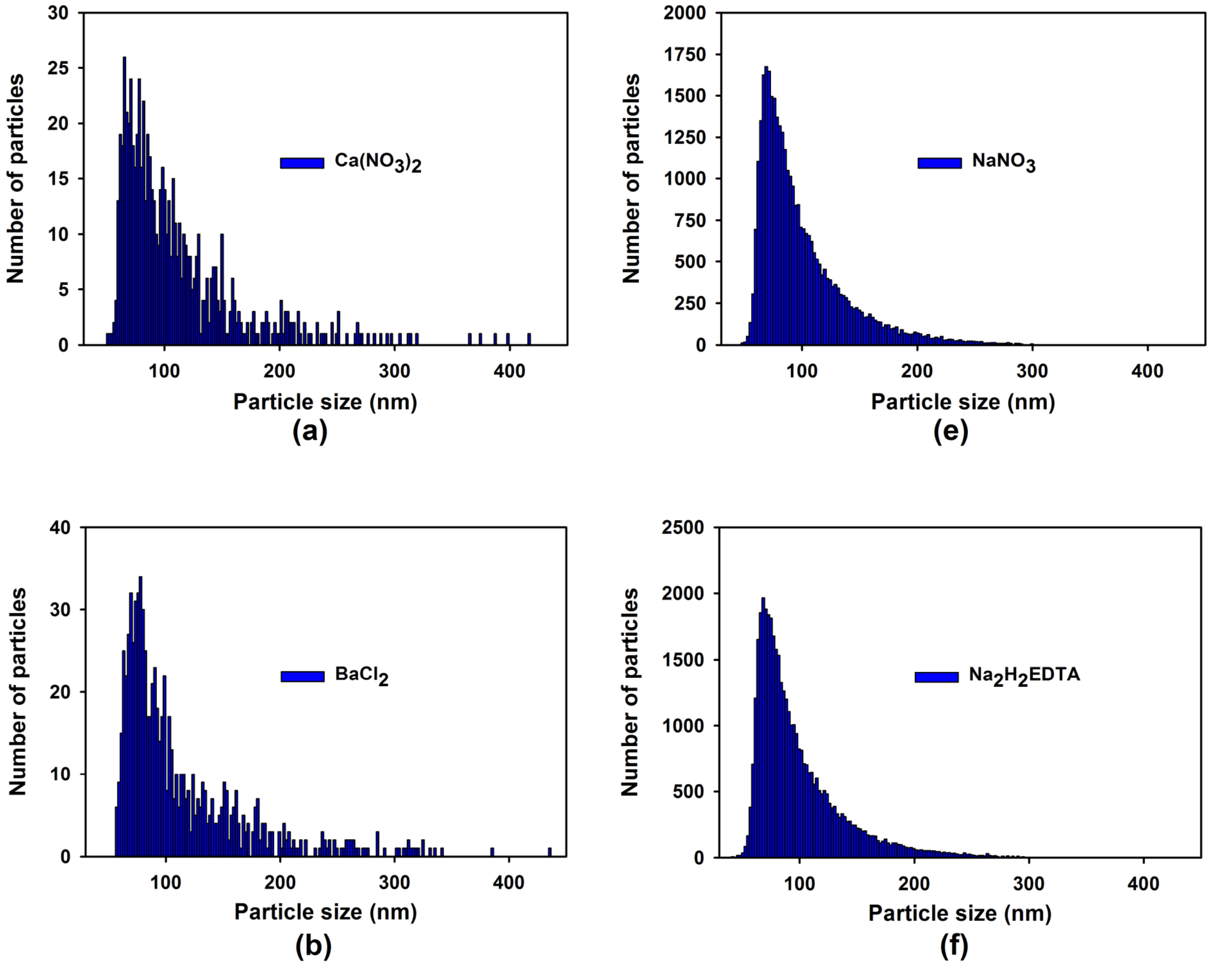

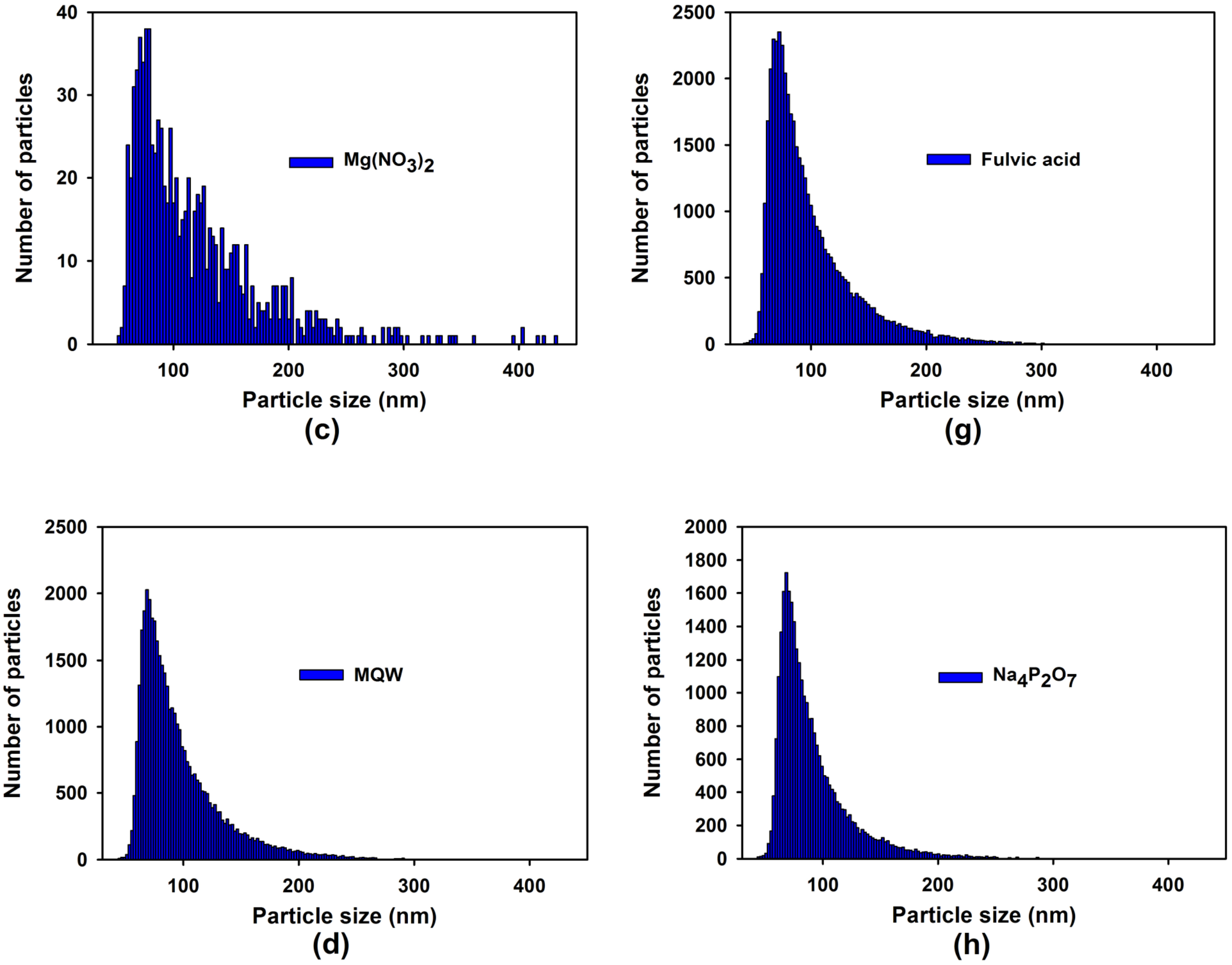

Figure S5. Particle size distribution of Si-containing NPs in the soil leachates obtained using different extractants: (a) $Ca(NO_3)_2$ (5 mmol L^{-1}), (b) $BaCl_2$ (5 mmol L^{-1}), (c) $Mg(NO_3)_2$ (5 mmol L^{-1}), (d) Milli-Q water, (e) $NaNO_3$ (5 mmol L^{-1}), (f) Na_2H_2EDTA (0.1 mmol L^{-1}), (g) FA (40 mg L^{-1}) and (h) $Na_4P_2O_7$ (5 mmol L^{-1}). The pH was adjusted to 6.0 for all extraction solutions prior to contact with the soil. The leachates were diluted 40000 times prior to SP-ICP-MS analysis, except in the case of $Na_4P_2O_7$ for which a dilution factor of 250000 times was necessary. Particle sizes correspond to equivalent diameters calculated under the assumption that the NP are spherical SiO_2.

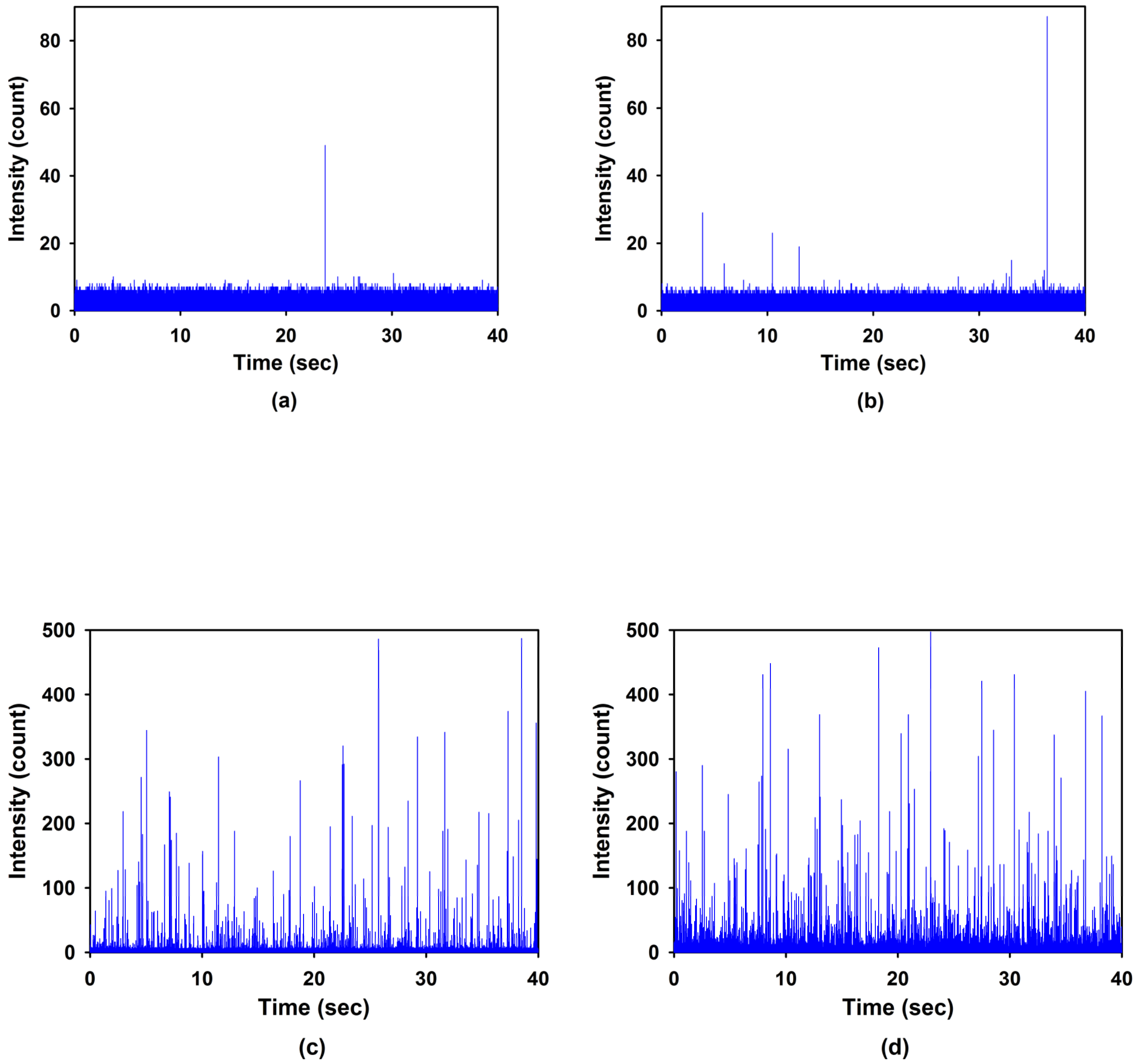

Figure S6. Raw data for SP ICP-MS analysis of ^{28}Si in a) Milli-Q water, b) solution of 40 mM $Na_4P_2O_7$ that was diluted 250000 times prior to analysis, c) diluted (250000x) soil leachate obtained using Milli-Q water, and d) diluted (250000x) soil leachate obtained with 40 mM $Na_4P_2O_7$. All dilutions were done with Milli-Q water.

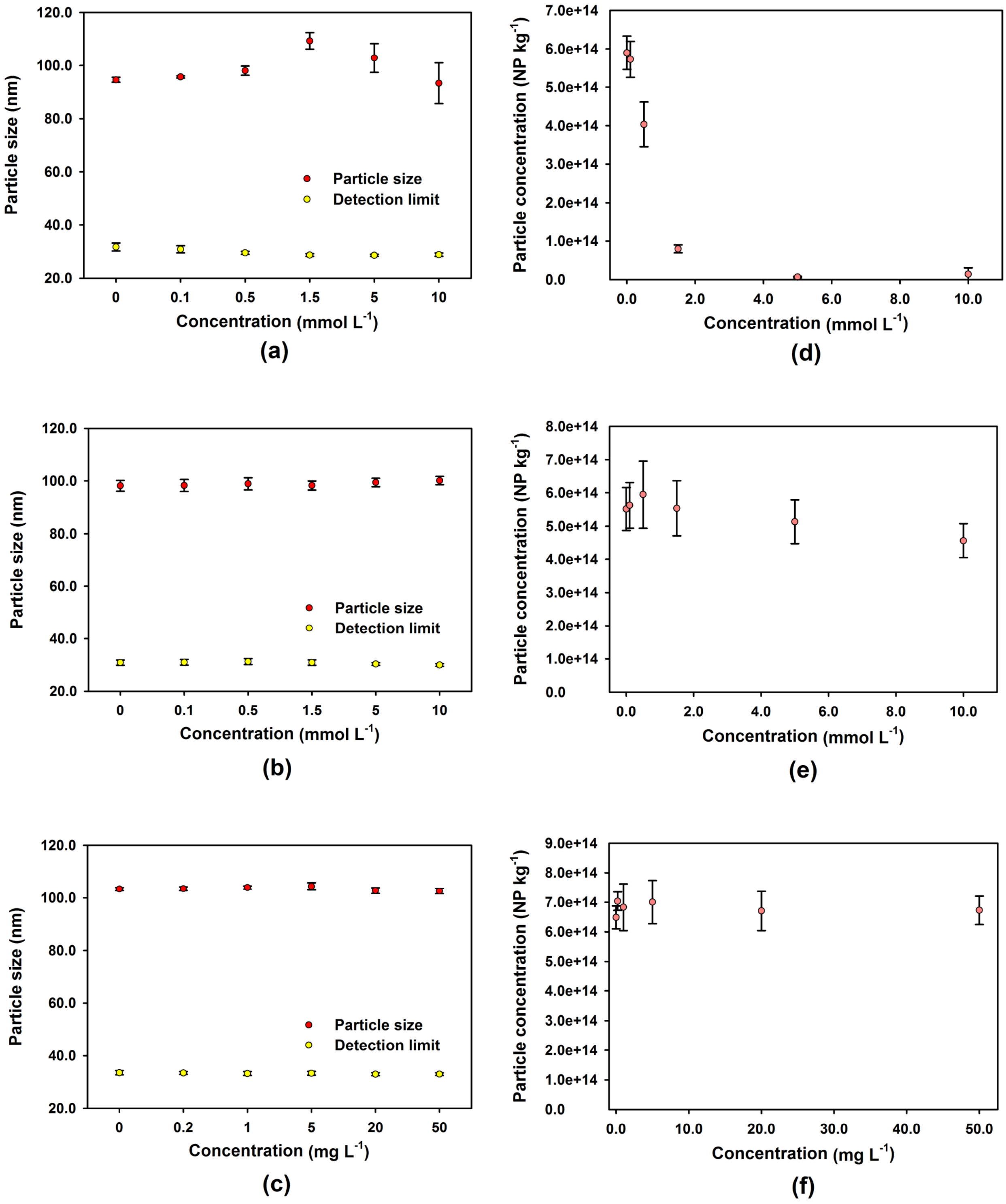

Figure S7. (a, b, c) size and (d, e, f) concentration of Si-containing NPs in the extraction solutions of a soil in contact (18 hours) with different extractants as a function of the concentration of (a, d) $Ca(NO_3)_2$, (b, e) $NaNO_3$, and (c, f) FA. The concentration of 0 represents the initial extraction using Milli-Q water. Particle sizes correspond to equivalent diameters calculated under the assumption that the NP are spherical SiO_2.

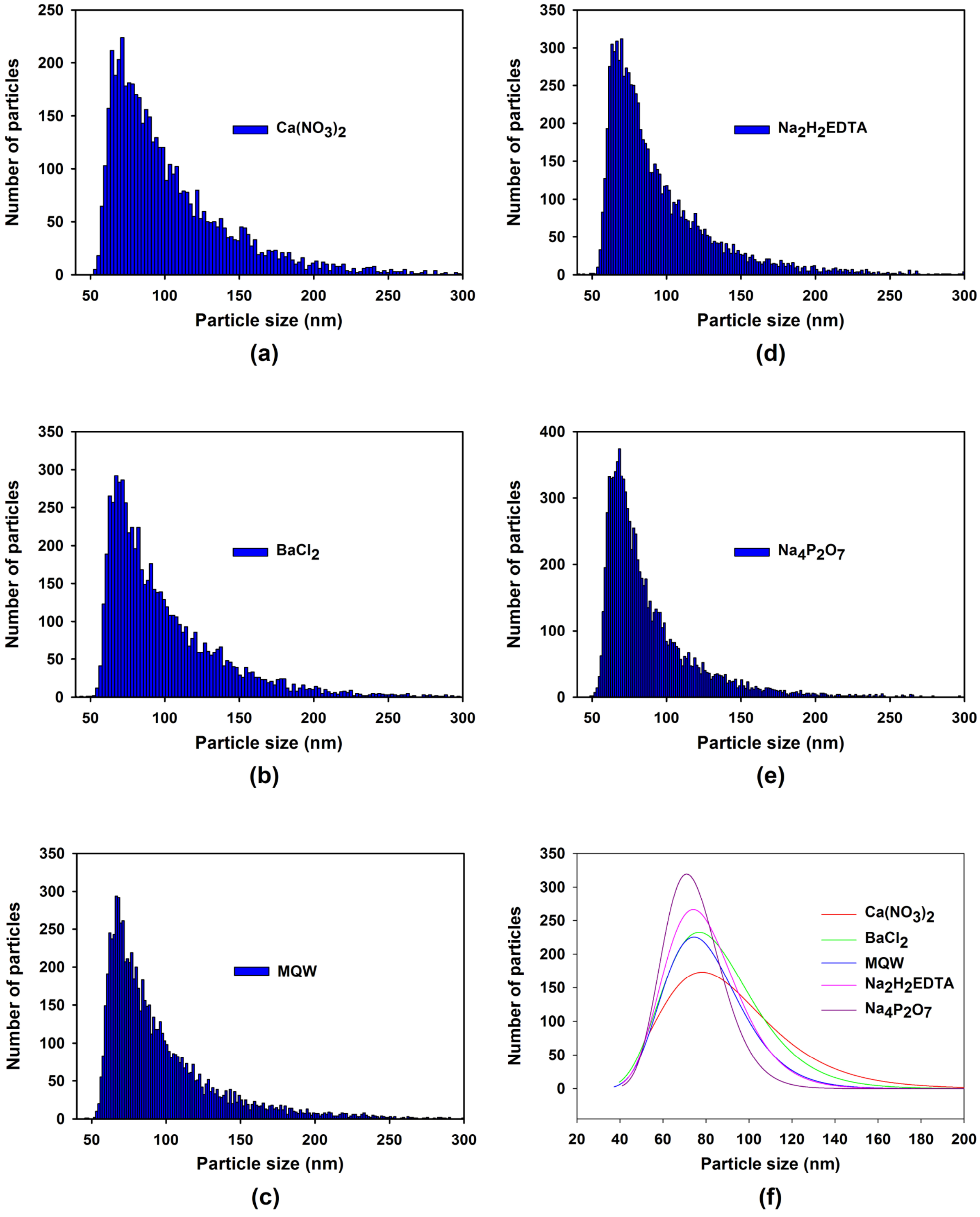

Figure S8. Particle size distribution of Si-containing NPs extracted from the soil using Milli-Q water, which were then suspended in different media: (a) $Ca(NO_3)_2$ (5 mmol L^{-1}), (b) $BaCl_2$ (5 mmol L^{-1}), (c) Milli-Q water, (d) Na_2H_2EDTA (0.1mmol L^{-1}) or (e) $Na_4P_2O_7$ (5 mmol L^{-1}). (f) Polynomial fitting the particle size histograms. The leachates were diluted 100000 times prior to SP-ICP-MS analysis. Particle sizes correspond to equivalent diameters calculated under the assumption that the NP are spherical SiO_2.

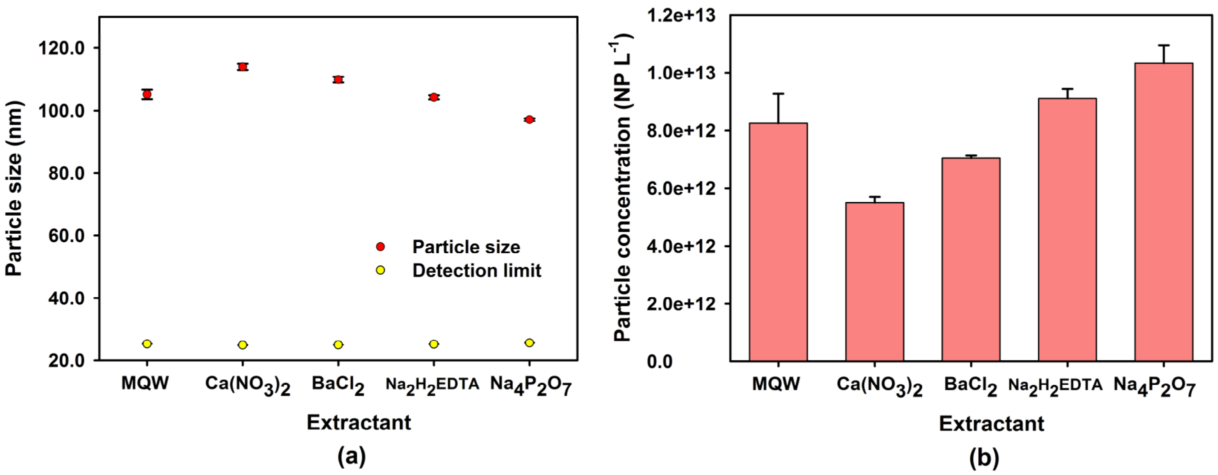

Figure S9. (a) Average size and (b) concentration of Si-containing NPs extracted from the soil using Milli-Q water, which were then suspended in different media: $Ca(NO_3)_2$ (5 mmol L^{-1}), $BaCl_2$ (5 mmol L^{-1}), $Na_4P_2O_7$ (5 mmol L^{-1}) or Na_2H_2EDTA (0.1 mmol L^{-1}). Particle sizes correspond to equivalent diameters calculated under the assumption that the NP are spherical SiO_2.

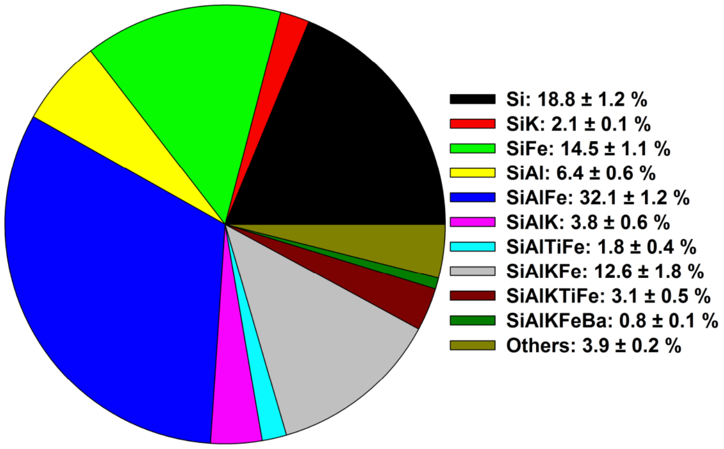

Figure S10. Proportion of each Si particle type detected in the soil leachate using pure water as the extractant and SP TOF-ICP-MS for detection. Leachates were diluted 100000 times prior to analysis. 854 particles were detected during an acquisition time of 92 sec. A dwell time of 76.8 µsec was employed.

References

1. Galhardi, J.A.; Wang, P.Y.; Bueno, V.; Ghoshal, S.; Gravel, V.; Wilkinson, K.J.; Bayen, S. Field evaluation of the potential effects of polymer and silica-based nanopesticides on strawberries and agricultural soils. *Environmental Science-Nano* **2022**, *9*, 3833-3843, doi:10.1039/d2en00329e.
